# Investigation on Hot Deformation Behavior and Microstructural Evolution of Al-Mg-Zn Aluminum Alloy via Uniaxial Isothermal Hot Compression Tests

**DOI:** 10.3390/ma18214903

**Published:** 2025-10-27

**Authors:** Fei He, Junzhe Huang, Kai Zhu, Xiwu Li, Kai Wen, Guanjun Gao, Lizhen Yan, Hongwei Yan, Zhihui Li, Yongan Zhang, Baiqing Xiong

**Affiliations:** 1 State Key Laboratory of Nonferrous Structural Materials, China GRINM Group Co., Ltd., Beijing 100088, China; 2 General Research Institute for Nonferrous Metals, Beijing 100088, China; 3 GRIMAT Engineering Institute Co., Ltd., Beijing 101407, China

**Keywords:** hot deformation behavior, microstructural evolution, high-magnesium content aluminum alloy, uniaxial hot compression tests, dynamic recrystallization

## Abstract

In this study, we investigated the hot deformation behavior and microstructural evolution of a novel high-magnesium-content (high-Mg) aluminum alloy, bridging the disciplines of material processing and physical metallurgy. Uniaxial hot compression tests were performed over the temperature range of 280~400 °C and strain rates of 0.001~10 s^−1^ to investigate its hot deformation behavior. The flow stress curves were systematically analyzed, and a constitutive model was developed to describe the thermo-mechanical response of the alloy. Microstructural evolution was characterized using scanning electron microscopy (SEM) and electron backscatter diffraction (EBSD). The results indicate that dynamic recovery serves as the dominant softening mechanism at lower deformation temperatures (≤320 °C). As the temperature increased to 400 °C, a significant rise in dynamic recrystallization was observed. Moreover, at 400 °C, higher strain rates led to the formation of abundant, network-like, mushroom-shaped dynamically recrystallized grains.

## 1. Introduction

Aluminum alloys, characterized by their low density, high strength, excellent corrosion resistance, fatigue performance, and formability, are one of the preferred materials for achieving lightweight design and manufacturing of structural components in fields such as aerospace, high-speed rail vehicles (HSRs), automotive engineering, power electronics, and others [1,2,3,4,5,6]. However, the properties of different types of aluminum alloys often result from their distinct processing methods. For instance, the 2xxx, 6xxx and 7xxx series alloys derive their strength primarily from T6 heat treatment (solution treatment and artificial aging), whereas other series, including many 5xxx alloys, rely on strain hardening during plastic deformation. Leveraging this principle, recent studies have found that by adding Zn elements to conventional 5xxx series aluminum alloys, novel Al-Mg-Zn alloys with compositions falling between traditional 5xxx and 7xxx series have been developed, exhibiting promising mechanical properties [7]. Microstructural analysis revealed that a large number of nanoscale T-Mg_32_(Al,Zn)_49_ precipitates, which have a significant strengthening effect, were extensively and dispersedly formed within the matrix of this new Al-Mg-Zn alloy in its T6 state—this is identified as a key factor contributing to the enhanced strength [8,9,10].

According to reference [11], it was first discovered in the 5xxx series alloys that the addition of Zn elements could significantly improve the stress corrosion cracking (SCC) resistance of Al-Mg alloys. Through investigations employing transmission electron microscopy (TEM) and energy-dispersive X-ray spectroscopy (EDS), it was revealed that T-Mg_32_(Al,Zn)_49_ precipitates distributed along grain boundaries play a crucial role in enhancing the alloy’s corrosion resistance, exhibiting superior SCC resistance compared to the β phase. Previous studies [12,13] have reported the effects of adding elements such as Zn, Cu, and Ag to Al-Mg alloys, along with different aging processes, on the aging hardening behavior of aluminum alloys. Results from hardness tests, tensile tests, and transmission electron microscopy (TEM) demonstrate that single-step artificial aging increases the peak-aged hardness by 58% in Zn-modified alloys, 56% in Zn-Cu-containing alloys, while trace additions of Cu and Ag accelerate the aging process. Additionally, in recent years, pertinent research institutions have conducted comprehensive investigations into the influence of varying Zn and Mg contents on stress corrosion cracking (SCC) and intergranular corrosion behaviors in the novel Al-Mg-Zn alloys [12,13,14,15].

The industrial manufacturing of aluminum components—such as thick plates, extrusions, and forgings—critically depends on the hot deformation process [16,17]. This complex procedure involves significant thermo-mechanical coupling, where applied stress induces both immediate plastic flow and concurrent microstructural evolution. Among these phenomena, the flow stress during the alloy hot deformation process serves as a critical indicator of its plastic formability and provides an essential basis for formulating optimal processing parameters. The primary method for characterizing the flow behavior of an alloy lies in establishing its constitutive relationship under the specific deformation conditions [18,19]. An accurate constitutive model not only enables researchers to comprehensively understand the deformation characteristics of the material, but also supplies reliable useful parameters for finite element simulation analyses tailored to industrial forming processes.

Compared to traditional alloys, the newly developed high-Mg content aluminum alloys exhibit a narrower hot plastic deformation range, inferior hot workability, and heightened sensitivity to thermal processing parameters, accompanied by a significant risk of cracking during deformation. Therefore, this study was undertaken with three primary objectives: (1) to characterize the flow stress behavior of this novel high-Mg alloy across a range of deformation temperatures and strain rates, (2) to establish an accurate constitutive model describing its hot deformation mechanics, and (3) to identify the dominant dynamic softening mechanisms operating during deformation. To achieve these goals, systematic investigations were conducted on the hot deformation behavior of this novel high-Mg aluminum alloy using single-pass uniaxial compression hot simulation experiments. In this study, those obtained flow stress curves under varying deformation temperatures and strain rates, established mathematical correlations among strain (ε), strain rate ($\dot{\varepsilon }$), and deformation temperature (*T*), and consequently was constructed a constitutive model specifically for this high-Mg aluminum alloy. Microstructure before and after deformation was characterized through the scanning electron microscopy (SEM) and the electron backscattered diffraction (EBSD) analyses, enabling detailed discussions on the dynamic softening mechanisms operating during alloy deformation.

## 2. Experimental

**Figure 1:**
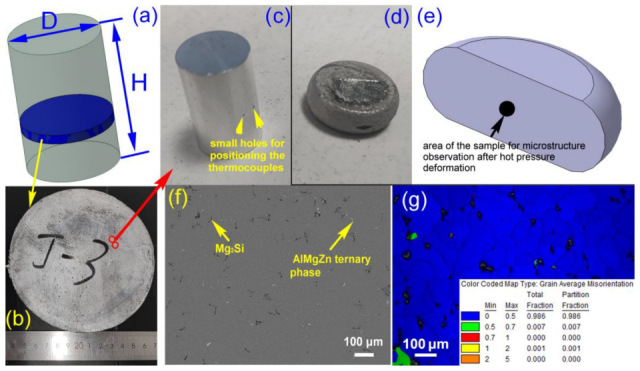
A schematic illustration of the as-cast billet and sampling positions on its cross-section (**a**), billet slice (**b**), specimen before hot simulation compression deformation (**c**), deformed specimen (**d**), schematic diagram indicating the observation locations for microstructural analysis after deformation (**e**), SEM micrograph and EDS point analysis results of the homogenized alloy (**f**), EBSD micrograph of the homogenized alloy (grain average misorientation map) (**g**).

The alloy used in this research was self-produced via melting and casting in the laboratory. Firstly, high-purity aluminum ingots and master alloys were weighed in precise proportions and melted in a laboratory-owned well-type electric resistance furnace for casting. Once the complete dissolution of all constituents was achieved, slag removal and degassing treatments were performed on the molten metal, followed by pouring into a water-cooled copper mold for solidification. Using the inductively coupled plasma-atomic emission spectrometry (ICP-AES) for the composition analysis, the approximate values of major alloying element contents (by mass percentage, wt%) of the alloy are 7.32% Mg, 2.74% Zn, and 0.48% Si, respectively. Subsequently, the as-cast billet underwent homogenization heat treatment, with a cross-sectional slice after treatment shown in Figure 1a,b. Thirdly, sufficient specimens were extracted from the D/4 radial position of the billet slices (marked by the red circles shown in Figure 1b) using wire electrical discharge machining (EDM), which were then machined into smooth-surfaced cylindrical samples (∅10 mm × 15 mm) through turning operations, achieving a surface roughness of Ra = 1.6 μm. Finally, two circular holes were drilled along the circumference of each cylindrical sample at the positions indicated in Figure 1c. It should be emphasized that a number of samples are required for robust hot simulation compression experiments; consequently, the red dashed box in Figure 1b is intended to represent one representative sampling location.

Uniaxial hot compression simulation experiments were conducted on a Gleeble-1500 testing machine(Dynamic Systems Inc., Albany, NJ, USA). Initially, thermocouple wires for temperature feedback and control were inserted into the circular holes shown in Figure 1c, while graphite sheets were affixed to both ends of the specimens to minimize friction between the sample surfaces and the machine grips. The specimens were then positioned between the grips of the thermomechanical simulator. According to the experimental protocol, samples were heated at a rate of 10 °C/s to temperatures ranging from 280 °C to 400 °C, held isothermally for 3 min to ensure homogeneous temperature distribution, and subsequently subjected to uniaxial compression deformation with strain rates varying from 0.001 s^−1^ to 10 s^−1^, achieving a total strain of 60%. During testing, the Gleeble-1500 system autonomously controlled temperature, strain magnitude, and strain rate parameters, while simultaneously recording stress–strain data. Upon completion of deformation, specimens were immediately quenched into the room-temperature water to preserve their high-temperature deformed microstructures, as illustrated in Figure 1d.

Post-water-quenched specimens were symmetrically sectioned along the compression axis for microstructural observation via electron backscattered diffraction (EBSD, EDAX Inc., Mahwah, NJ, USA), with the characterized region depicted in Figure 1e. The preparation sequence involved: initial grinding using successive grades of water-abrasive papers (#400, #600, #800, #1000, and #2000); subsequent polishing with 1 μm diamond suspension; final electrolytic etching at room temperature using a solution comprising 10% HClO_4_ and 90% C_2_H_5_OH, applied at 30 V for 10 s. For reference, as-homogenized but undeformed baseline samples were similarly processed—sectioned longitudinally, subjected to identical mechanical grinding/polishing protocols—prior to undergoing microstructural examination using a JSM-7001F field emission gun scanning electron microscope (FE-SEM, JEOL Corporation, Tokyo, Japan) and energy dispersive spectrometry (EDS) analysis conducted with an Octane Super EDS detector (EDAX Inc., Mahwah, NJ, USA).

## 3. Results and Discussion

### 3.1. Single-Pass Hot Compression Flow Behavior of Alloys

**Figure 2:**
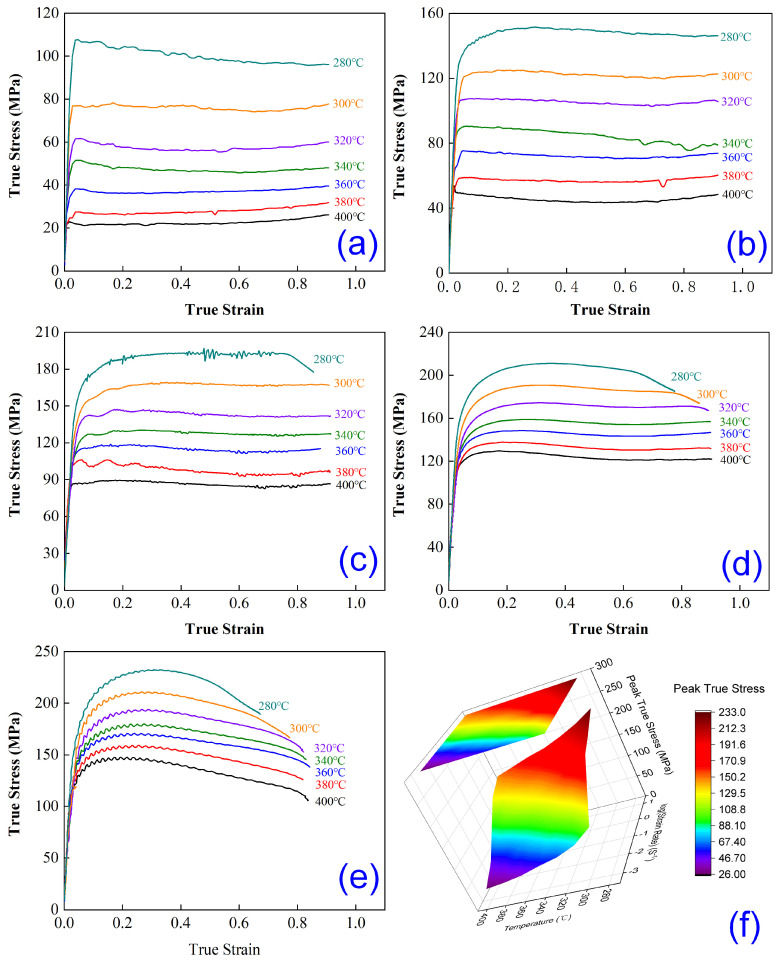
True stress−strain curves and peak stress maps of the novel alloy deformed under different conditions: (**a**) 0.001 s^−1^, (**b**) 0.01 s^−1^, (**c**) 0.1 s^−1^, (**d**) 1 s^−1^, (**e**) 10 s^−1^, and (**f**) peak stress maps.

Figure 2 presents the measured true stress–strain curves of the newly developed high-magnesium-content (Mg) aluminum alloy after homogenization treatment, deformed under varying temperatures (280~400 °C) and strain rates (0.001~10 s^−1^), with actual strains all exceeding 0.7. As evident from the curves, during the initial stage of hot compression deformation, the flow stress rapidly increases with increasing strain across all tested conditions, reaching a peak value. Specifically, the following is true:

- At deformation temperatures of 300~400 °C and strain rates of 0.001~1 s^−1^, as well as at 280 °C with strain rates of 0.001~0.01 s^−1^, the flow stress slightly decreases after peaking before stabilizing.
- At 280 °C with strain rates of 0.1~10 s^−1^, the flow stress declines gradually after reaching its peak.
- At 300~400 °C and a strain rate of 10 s^−1^, the flow stress exhibits a pronounced decreasing trend post-peak.

These observations demonstrate that both deformation temperature and strain rate critically influence the flow stress behavior of the alloy, attributed to the competitive interplay between work hardening and dynamic softening mechanisms during hot deformation. Notably, as illustrated in Figure 2f, at a constant strain rate of 0.001 s^−1^, elevating the deformation temperature from 280 °C to 400 °C reduces the peak stress dramatically from 107.79 MPa to 26.19 MPa (~76% reduction). This significant drop stems from the thermally activated nature of plastic deformation: higher temperatures enhance atomic kinetic energy and lattice vibration amplitude, facilitating vacancy migration, edge dislocation climbing, and screw dislocation cross-glide. These processes promote dislocation annihilation, reduce the dislocation density, and improve ductility, thereby lowering the peak flow stress. Additionally, intensified thermal activation accelerates dynamic recrystallization, enhancing nucleation and grain boundary migration rates, which consume dislocations and activate intragranular slip systems, collectively diminishing deformation resistance. Conversely, at a fixed temperature of 400 °C, increasing the strain rate from 0.001 s^−1^ to 10 s^−1^ elevates the peak stress from 26.19 MPa to 147.76 MPa (~82% increase). Higher strain rates shorten the time available for dynamic softening, leading to rapid dislocation accumulation, entanglement, and pile-up. Consequently, the work hardening dominates under high-strain-rate conditions, resulting in substantially higher flow stress levels.

Based on the flow stress curves of the alloy obtained under different compression conditions mentioned above, constitutive equations can be established to determine the relationship between deformation temperature, strain rate, and flow stress. Until now, researchers have developed various constitutive models, among which the Arrhenius equation is the most representative and has been extensively used to describe the interrelationship between the flow behavior of metallic materials and deformation parameters [20,21,22,23]. Its mathematical expression is as follows:

(1) $$Z=\dot{\varepsilon }\exp\left(Q_{c}/\left(RT\right)\right)$$

where *Z* is the Zener–Hollomon parameter, $\dot{\varepsilon }$ is the strain rate, *Q_c_* is the activation energy for hot deformation, *R* is the universal gas constant, *T* is the compression deformation temperature of the alloy.

The expression form of Equation (1) varies with different flow stress levels. When the flow stress level is relatively low, Equation (1) takes the following form:

(2) $$\dot{\varepsilon }=A_{1}\sigma^{n_{1}}\exp\left(-Q_{c}/\left(RT\right)\right),\;\alpha \sigma <0.8$$

when the flow stress level is relatively high, Equation (1) takes the following form:

(3) $$\dot{\varepsilon }=A_{2}\exp\left(\beta \sigma \right)\exp\left(-Q_{c}/\left(RT\right)\right),\;\alpha \sigma >1.2$$

More generally, for all the flow stress levels, the expression of Equation (1) is as follows:

(4) $$\dot{\varepsilon }=A{\left[\sinh\left(\alpha \sigma \right)\right]}^{n}\exp\left(-Q_{c}/\left(RT\right)\right)$$

In the above equations, *A*, *A*_1_, *A*_2_, *n*, *n*_1_, *α*, and *β* are material constants; *σ* is the flow stress; and *α = β/n*_1_.

Taking the logarithm of both sides of Equations (2) and (3), it is found that:

(5) $$\ln\dot{\varepsilon }=\ln A_{1}+n_{1}\ln\sigma -\frac{Q_{c}}{RT}$$

(6) $$\ln\dot{\varepsilon }=\ln A_{2}+\beta \sigma -\frac{Q_{c}}{RT}$$

**Figure 3:**
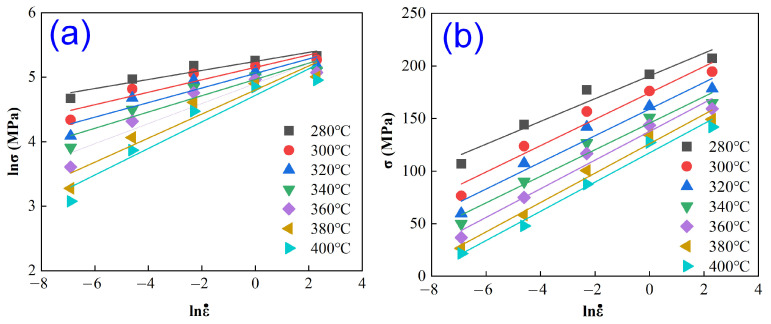
Relationship curves of $\ln\dot{\varepsilon }$ vs. lnσ (**a**) and $\ln\dot{\varepsilon }$ vs. σ (**b**).

By substituting the flow stress values at the strain of 0.1, along with deformation temperature and strain rate, into Equations (5) and (6), linear fitting was performed to establish the relationships of $\ln\dot{\varepsilon }$ vs. lnσ and $\ln\dot{\varepsilon }$ vs. σ under different deformation conditions, as presented in Figure 3. The average values derived from the fitted curves in the figure yielded the parameters *n*_1_ = 0.1363 and *β* = 13.3011 MPa^−1^. Subsequently, α was calculated as *α* = *β*/*n*_1_ = 0.0102 MPa^−1^.

Taking the logarithm of both sides of Equations (1) and (4), it is found that:

(7) $$\ln Z=\ln\dot{\varepsilon }+\frac{Q_{c}}{RT},$$

(8) $$\ln\dot{\varepsilon }=\ln A+n\ln\left[\sinh\left(\alpha \sigma \right)\right]-\frac{Q_{c}}{RT}$$

Further rearranging Equations (7) and (8) yields:

(9) $$\ln Z=\ln A+n\ln\left[\sinh\left(\alpha \sigma \right)\right]$$

Under the given deformation temperature and strain rate conditions, partial differentiation of Equation (7) gives:

(10) $$\frac{1}{n}={\left\{\frac{\partial \ln\left[\sinh\left(\alpha \sigma \right)\right]}{\partial \ln\dot{\varepsilon }}\right\}}_{T},$$

(11) $$\frac{Q_{c}}{nR}={\left\{\frac{\partial \ln\left[\sinh\left(\alpha \sigma \right)\right]}{\partial \left(1/T\right)}\right\}}_{\dot{\varepsilon }}.$$

Let $Q_{c}/nR=M$; then, Equation (11) can be written as follows:

(12) $$Q_{c}=R\cdot n\cdot M$$

**Figure 4:**
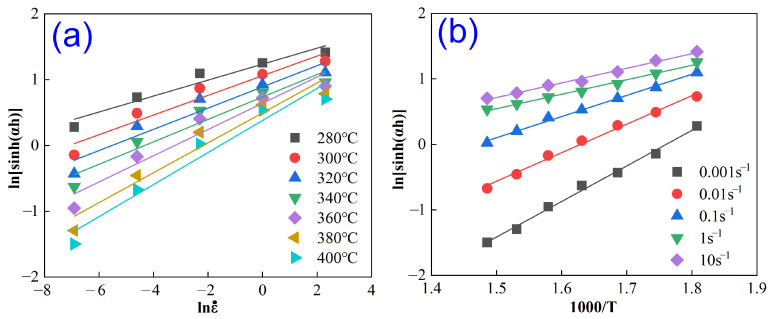
Relationship plots of $\ln\left[\sinh\left(\alpha \sigma \right)\right]$ vs. $\ln\dot{\varepsilon }$ (**a**) and $\ln\left[\sinh\left(\alpha \sigma \right)\right]$ vs. $T^{-1}$ (**b**).

Therefore, Equations (10) and (11) correspond to the slopes of $\ln\left[\sinh\left(\alpha \sigma \right)\right]$ vs. $\ln\dot{\varepsilon }$ and $\ln\left[\sinh\left(\alpha \sigma \right)\right]$ vs. $T^{-1}$, respectively. Substituting the relevant experimental data into Equation (8) and performing the linear fitting yields the results plotted in Figure 4. Further calculations yield *n* = 5.4983 and *M* = 3.489, thereby determining the activation energy for hot deformation as *Q_c_* = 159.50 kJ/mol. 

**Figure 5:**
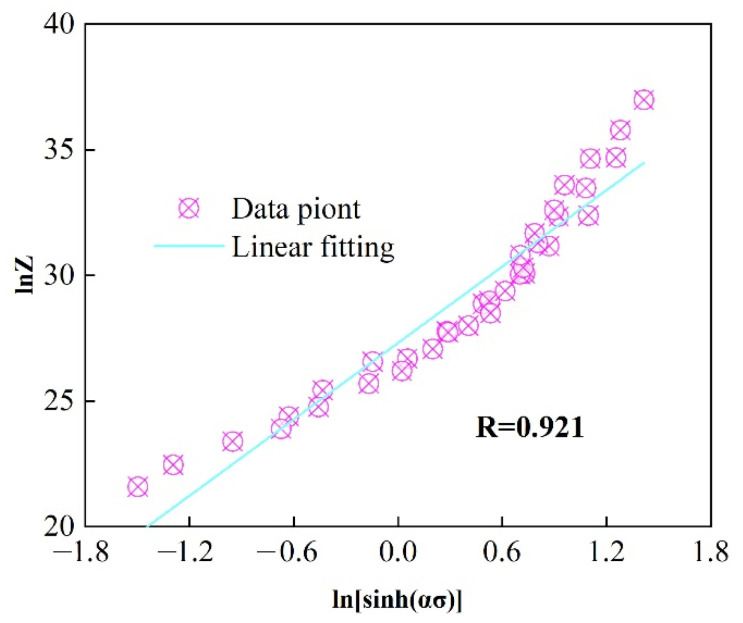
Relationship plot of lnZ vs. $\ln\left[\sinh\left(\alpha \sigma \right)\right]$.

Then, substituting the obtained *Q_c_* value into Equations (7) and (9), and performing the linear fitting, the obtained results are presented in Figure 5. Further calculations yield *n* = 5.0637 and ln*A* = 27.3073. Notably, the calculated *n* value closely aligns with the previously determined result. The correlation coefficient *R* = 0.921 indicates a high degree of linearity, demonstrating that the established constitutive model reliably predicts the flow stress evolution behavior of the novel alloy during hot deformation.

Substituting the values calculated above into Equation (4), the constitutive equation for the novel alloy can be depicted as follows:

(13) $$\dot{\varepsilon }=e^{27.3073}{\left[\sinh\left(0.0102\sigma \right)\right]}^{5.0637}\exp\left(-\frac{159.5}{RT}\right)$$

### 3.2. Effect of Deformation Temperature on the Microstructure of the Alloy

Here, it should be firstly noted that the SEM micrograph of the homogenized alloy shown in Figure 1f reveals a large number of fine, uniformly and dispersedly distributed AlMgZn ternary phases (appearing as white particles) [7]. These particles mainly precipitated during air cooling after the ingot underwent homogenization heat treatment. Additionally, undissolved Mg_2_Si phases predominantly distributed at triple grain boundaries are observed (due to its relatively high dissolution temperature) [24]. The EBSD analysis results presented in Figure 1g indicate that complete recrystallization occurred in the grains following homogenization treatment of the ingot, and those grains are uniform in size and equiaxed in shape. Regarding the EBSD findings, it is generally accepted that grains with an average intragranular orientation value spread within 0~1 are classified as recrystallized grains, those within 1~2 as recovered grains, and those within 2~5 as deformed grains [25].

**Figure 6:**
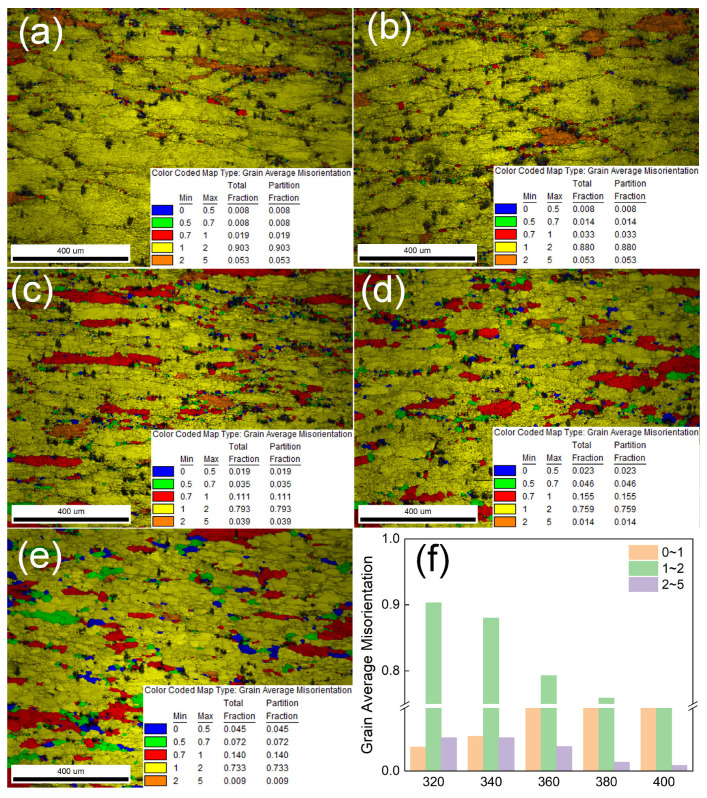
Grain average misorientation maps of the novel alloy deformed under the strain rate of 0.001 s^−1^ at different temperatures: (**a**) 320 °C, (**b**) 340 °C, (**c**) 360 °C, (**d**) 380 °C, (**e**) 400 °C, and (**f**) a bar chart of the statistical results.

Furthermore, Figure 6 displays the EBSD characterization results (grain average misorientation map) of the novel alloy after hot compression under conditions of strain ε = 0.9, strain rate $\dot{\varepsilon }$ = 0.001 s^−1^, and deformation temperatures ranging from 320 °C to 400 °C. As observed in the figures, grains within the alloy are universally flattened and elongated perpendicular to the specimen height, across all the tested conditions. Notably, at a deformation temperature of 320 °C, recovered grains dominate the microstructure, yet fine dynamically recrystallized grains begin to emerge locally at prior grain boundaries—a deviation from conventional aluminum alloys. Literature indicates that traditional Al alloys typically undergo minimal recrystallization at lower deformation temperatures, favoring dynamic recovery instead. With increasing deformation temperature, accelerated atom diffusion enhances dynamic recrystallization nucleation at high-energy grain boundaries. Elevated temperatures intensify thermal activation, expediting dynamic recrystallization and reducing its critical strain threshold. At the temperature of 400 °C, pronounced dynamic recrystallization manifests as denser recrystallized grains near original boundaries, accompanied by grain growth (evident in Figure 6e). The heightened thermal activity facilitates dislocation motion via climb and cross-glide, promoting transformation of subgrain structures into high-angle grain boundaries. Simultaneously, accelerated grain boundary migration drives grain coarsening. In summary, at constant strain rate, deformation temperature critically governs microstructural evolution: lower temperatures favor dynamic recovery, while higher temperatures progressively increase the proportion of dynamically recrystallized regions.

### 3.3. Effect of Strain Rates on the Microstructure of the Alloy

**Figure 7:**
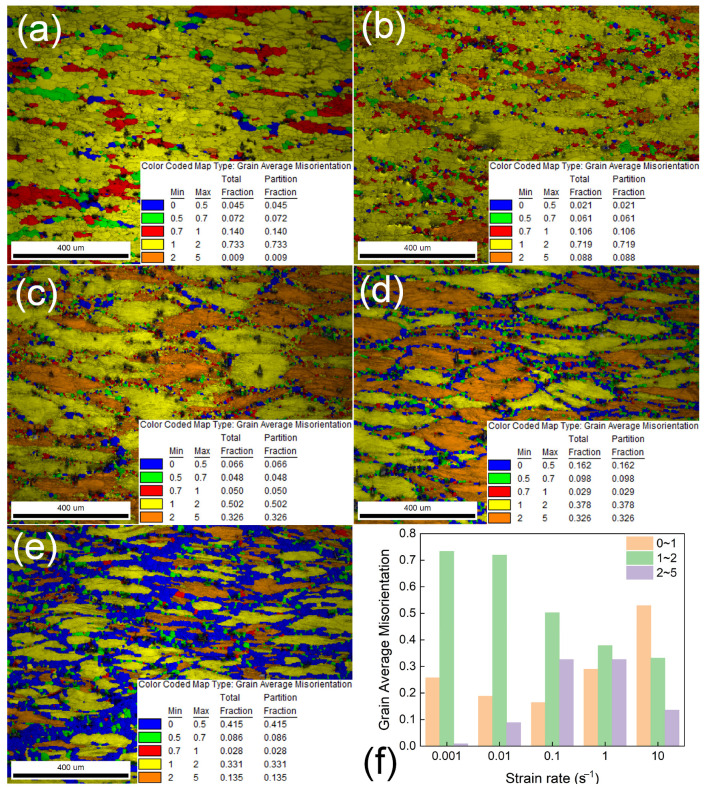
Grain average misorientation maps of the novel alloy deformed at 400 °C under different strain rates: (**a**) 0.001 s^−1^, (**b**) 0.01 s^−1^, (**c**) 0.1 s^−1^, (**d**) 1 s^−1^, (**e**) 10 s^−1^, and (**f**) a bar chart of the statistical results.

Figure 7 presents the EBSD characterization results of the novel alloy after compression at 400 °C, strain = 0.9, and strain rates ranging from 0.001 s^−1^ to 10 s^−1^. Consistent with prior observations, grains are universally flattened and elongated perpendicular to the specimen height across all the tested conditions. At a strain rate of 0.001 s^−1^ (shown in Figure 7a), dynamically recrystallized grains form at both original grain boundaries and within grain interiors, with localized grain growth evident. The extended deformation duration at this low strain rate enables dislocation motion via cross-glide and climb, facilitating substructure-to-high-angle-boundary transformation. Prolonged processing also allows sufficient time for grain boundary migration, enabling new grains to grow to a discernible size. As the strain rate increases (0.01~0.1 s^−1^), dynamic recrystallization predominantly occurs at original grain boundaries. Shortened deformation times reduce opportunities for dynamic softening, resulting in fewer recrystallized grains. Further increasing the strain rate (1~10 s^−1^) leads to a notable increase in both the quantity and size of dynamically recrystallized grains (shown in Figure 7e). The high magnesium content significantly elevates the alloy’s strength and deformation resistance, intensifying the energy dissipation and localized heating—particularly at defect-concentrated regions like grain boundaries. Under the high-strain-rate conditions, rapid dislocation proliferation occurs, while dynamic recovery mechanisms (dislocation climb and annihilation, etc.) are suppressed due to limited time. Dislocation pile-ups at grain boundaries generate stress concentrations, serving as preferential nucleation sites for dynamic recrystallization. Localized temperatures reaching or exceeding the critical threshold for dynamic recrystallization provide thermodynamic driving force for boundary nucleation.

Based on the above research results, it can be seen that even under relatively low deformation temperature (320 °C) and low strain rate (0.001 s^−1^), dynamic recrystallization occurred in this novel high-magnesium (Mg) content aluminum alloy during hot simulation compression deformation, which differs from conventional aluminum alloys [18,26]. It is well known that stacking fault energy (SFE) is a critical factor influencing the transition between the dynamic recovery and the dynamic recrystallization during alloy hot deformation [27,28]. Alloys with higher SFE exhibit easier dislocation slip and climb during deformation, resulting in lower stored deformation energy and microstructures dominated by recovered grains post-deformation. Conversely, alloys with lower SFE impede dislocation slip/climb, leading to localized dislocation tangle/pile-ups, elevated driving force for recrystallization, and ultimately microstructures predominantly composed of dynamically recrystallized grains [29]. Previous studies indicate that adding Mg reduces an aluminum alloy’s SFE [30], and this reduction increases the difficulty of dislocation slip/climb, significantly raising local stored energy and promoting dynamic recrystallization. Furthermore, the high Mg content in the investigated alloy leads to substantial solid-solution strengthening by Mg atoms, which tend to segregate near high-energy dislocation lines, forming Cottrell atmospheres that pin dislocation motion. Consequently, this directly leads to pronounced discrepancies in stress and strain distribution between the grain boundaries and the interior regions of grains. The grain boundary locations transform into high-energy zones due to extensive dislocation entanglement and pile-up phenomena. At these high-energy sites, the dense dislocation tangle structures undergo local polygonization first, subsequently transforming into recrystallized grains. Correspondingly, this effect becomes more pronounced under combined conditions of 400 °C deformation temperature and high strain rates. Higher strain rates accelerate dislocation multiplication at grain boundaries, creating regions with ultra-high dislocation density. Such dislocation dynamics, coupled with elevated deformation temperatures, facilitate dislocation rearrangement processes at grain boundaries, ultimately leading to a substantially higher volume fraction of dynamically recrystallized microstructures.

## 4. Conclusions

The current study was focused on the hot deformation behavior and microstructural evolution of a novel high-Mg-content aluminum alloy during hot compression. The constitutive equation was established, and the effects of deformation temperature and strain rate on the microstructure of the alloy were investigated. The key findings can be summarized as follows:

Under deformation temperatures of 280~400 °C and strain rates of 0.001~10 s^−1^, the material constants of the alloy were determined: A = e^27.3073^, activation energy for deformation *Q_c_* = 159.50 kJ/mol, *n* = 5.0637, and *α* = 0.0102 MPa^−1^. Therefore, the Arrhenius constitutive equation for the alloy is expressed as:

$$\dot{\varepsilon }=e^{27.3073}{\left[\sinh\left(0.0102\sigma \right)\right]}^{5.0637}\exp\left(-\frac{159.5}{RT}\right)$$

During the hot simulation compression process, deformation parameters exert a significant influence on the softening behavior of this novel aluminum alloy. At lower deformation temperatures (≤320 °C), the softening mechanism is dominated by dynamic recovery with secondary dynamic recrystallization; when the deformation temperature rises to 400 °C, the proportion of dynamically recrystallized microstructure within the alloy increases significantly. Under identical deformation conditions (400 °C), elevating the strain rate leads to a substantial increase in the fraction of dynamically recrystallized constituents.

Notably, due to the high magnesium content in this novel alloy—which results in a lower stacking fault energy, compared to conventional aluminum alloys—dynamic recrystallization persists even under relatively low deformation temperature (320 °C) and minimal strain rate (0.001 s^−1^) conditions during hot compression.

## Data Availability

The original contributions presented in this study are included in the article. Further inquiries can be directed to the corresponding authors.
